# Cystic tumor of the liver without ovarian-like stroma or bile duct communication: two case reports and a review of the literature

**DOI:** 10.1186/1477-7819-12-229

**Published:** 2014-07-21

**Authors:** Norihiro Kishida, Masahiro Shinoda, Yohei Masugi, Osamu Itano, Yoko Fujii-Nishimura, Akihisa Ueno, Minoru Kitago, Taizo Hibi, Yuta Abe, Hiroshi Yagi, Akihiro Tanimoto, Minoru Tanabe, Michiie Sakamaoto, Yuko Kitagawa

**Affiliations:** 1 Department of Surgery, Keio University, School of Medicine, 35 Shinanomachi, Shinjuku-ku, Tokyo 160-8582, Japan; 2 Department of Pathology, Keio University, School of Medicine, 35 Shinanomachi, Shinjuku-ku, Tokyo 160-8582, Japan; 3 Department of Diagnostic Radiology, Keio University, School of Medicine, 35 Shinanomachi, Shinjuku-ku, Tokyo 160-8582, Japan; 4 Department of Surgery, Tokyo Medical and Dental University, 1-5-45 Yushima, Bunkyo-ku, Tokyo 113-8519, Japan

**Keywords:** Cystic neoplasm, Liver, Ovarian-like stroma, Bile duct communication, Mucinous cystic neoplasm, Intraductal papillary neoplasm of bile duct

## Abstract

We report two cases of cystic neoplasm of the liver with mucinous epithelium in which both ovarian-like stroma and bile duct communication were absent. The first case was a 41-year-old woman. She underwent right trisegmentectomy due to a multilocular cystic lesion, 15 cm in diameter, with papillary nodular components in the medial segment and right lobe. Histologically, arborizing papillae were seen in the papillary lesion. The constituent neoplastic cells had sufficient cytoarchitectural atypia to be classified as high-grade dysplasia. The second case was a 60-year-old woman. She underwent left lobectomy due to a unilocular cystic lesion, 17 cm in diameter, in the left lobe. Histologically, the cyst wall was lined by low columnar epithelia with slight cellular atypia. In both cases, neither ovarian-like stroma nor bile duct communications were found throughout the resected specimen. According to the most recent World Health Organization (WHO) classification in 2010, cystic tumors of the liver with mucinous epithelium are classified as mucinous cystic neoplasms when ovarian-like stromata are found, and as intraductal papillary neoplasm of bile duct when bile duct communication exists. Therefore, we diagnosed the cystic tumors as ‘biliary cystadenoma’ according to the past WHO classification scheme from 2000. We believe that the combined absence of both ovarian-like stroma and bile duct communication is possible in mucinous cystic tumors of the liver. Herein, we have described the clinicopathologic features of the two cases and reviewed past cases in the literature.

## Background

The disease entity ‘biliary cystadenoma/adenocarcinoma’ was described in the World Health Organization (WHO) Classification of Tumors 3^rd^ Edition, published in 2000 [1]. The disease is rare, usually slow-growing, occurs predominantly in middle-aged women, and is characterized by multilocular cystic tumors filled with mucinous fluid. Microscopically, a single layer of mucin-secreting cells lines the cyst wall. However, the diagnostic criteria for biliary cystadenoma/cystadenocarcinoma have been vague. Cases of biliary cystadenoma/cystadenocarcinoma lacking ovarian-like stroma (OS) have been reported, suggesting that the diagnosis of cystadenoma or cystadenocarcinoma was not necessarily made based on the presence of OS [2,3]. Given this confusion, the latest WHO Classification of Tumors for the digestive system, published in 2010, proposed that the disease entity previously designated as ‘biliary cystadenoma/adenocarcinoma’ should instead be classified as either mucinous cystic neoplasm (MCN) or intraductal papillary neoplasm of bile duct (IPN-B) depending on the presence of OS and bile duct communication (BDC), respectively [4]. Herein, we report two cases of cystic neoplasm of the liver with mucinous epithelium in which both OS and BDC were absent, and which could not be clearly classified as either MCN or IPN-B. We encountered the first case after publication of the WHO classification system in 2010. The encounter with this case led us to survey our past cases that had been diagnosed as biliary cystadenoma or biliary cystadenocarcinoma according to the WHO classification system in 2000. We pathologically re-examined specimens of the past cases and identified another case without either OS or BDC. We report this past case as the second of the two case reports. We also reviewed past cases in the literature and discuss problems regarding the diagnostic criteria for cystic neoplasms of the liver in the absence of OS and BDC.

## Case presentation

### Case 1

**Figure 1:**
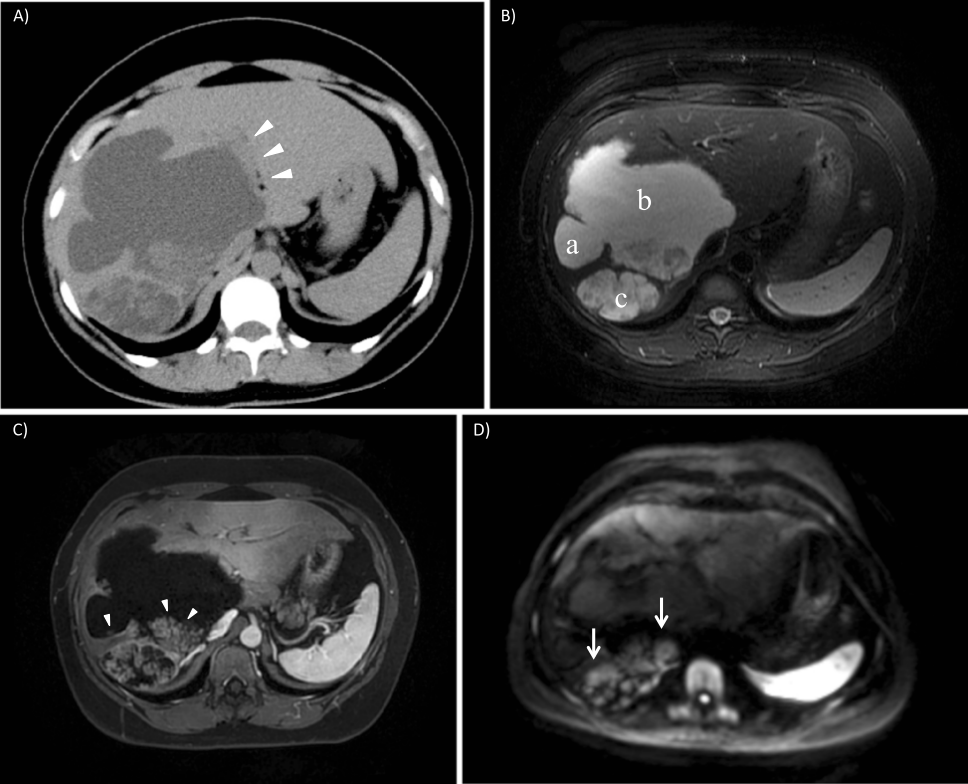
**Preoperative image findings (case 1). (A)** Computed tomography without contrast enhancement. A cystic lesion, 15 cm in diameter, is seen in the medial segment and right lobe. The lesion is multilocular, consisting of several separated lesions. The tumor is adjacent to the right side of the umbilical portion of the portal vein (umbilical portion is indicated by arrow heads). **(B)** T2-weighted, **(C)** contrast-enhanced, and **(D)** diffusion-weighted magnetic resonance imaging. T2-weighted image showed the cystic lesion in the medial segment and right lobe. On the section presented here, three separated lesions are seen (indicated by a, b, and c). Contrast enhancement showed that each separated lesion contained multiple papillary nodular components with enhancement. Cystic lesion (a) contains a tiny nodule inside (indicated by an arrowhead), cystic lesion (b) has a papillary nodular component in its posterior side (indicated by arrowheads), and lesion (c) was filled with papillary nodular components. A diffusion-weighted image showed diffusion restriction in the nodular components in the lesion of (b) and (c) (indicated by arrows).

A 41-year-old Japanese woman had undergone laparoscopic deroofing due to a liver cyst in the right lobe in 2009 at another hospital. After the operation, the cystic lesion could still be confirmed by ultrasonography, and the patient subsequently underwent follow-up for blood analysis and ultrasonography every six months for three years. Recent ultrasonography revealed an increase in the size of the cystic lesion. The patient was referred to our hospital for further examination in 2012. Since the patient had a medical history of iodine hypersensitivity, we could not employ contrast-enhanced computed tomography and endoscopic retrograde cholangiography (ERC) for the examinations. Computed tomography without contrast enhancement revealed a multilocular cystic lesion, 15 cm in diameter, in the medial segment and right lobe (Figure 1A). T2-weighted magnetic resonance imaging also clearly revealed the cystic lesion (Figure 1B). Contrast-enhanced magnetic resonance imaging showed that the lesion contained multiple papillary nodular components with enhancement (Figure 1C). Diffusion-weighted magnetic resonance imaging showed diffusion restriction in the nodular components (Figure 1D). Ultrasonography with Sonazoid™ (Daiichi-Sankyo, Tokyo, Japan) revealed an enhancement corresponding to the solid component that was evident by diffusion-weighted magnetic resonance imaging. No evidence of metastases to the lymph nodes or other organs was found in the preoperative images. The extrahepatic bile duct was not dilated in the images. Aspartate aminotransferase, alanine aminotransferase, alkaline phosphatase, gamma-glutamyltransferase, albumin, and total bilirubin levels were all within normal limits. Serology tests were negative for hepatitis B and C viruses. The serum levels of carcinoembryonic antigen and CA19-9 were within normal limits. According to the pathology report from the patient’s previous hospital, the resected cyst wall was lined by simple columnar epithelium with papillary change, no malignant changes were evident, and OS was absent in the specimen. The diagnosis in the report was cystadenoma. From the information contained in the previous report and the findings from imaging at our hospital, we could not diagnose the cystic lesion as either MCN or IPN-B, but we suspected that the solid component in the cystic lesion had a malignancy and performed right trisegmentectomy. Ten days after surgery, she developed bile leakage and underwent endoscopic retrograde nasobiliary drainage. The postoperative course was otherwise uneventful and the patient was discharged on postoperative day 64. The patient is still alive at 19 months postoperatively.

**Figure 2:**
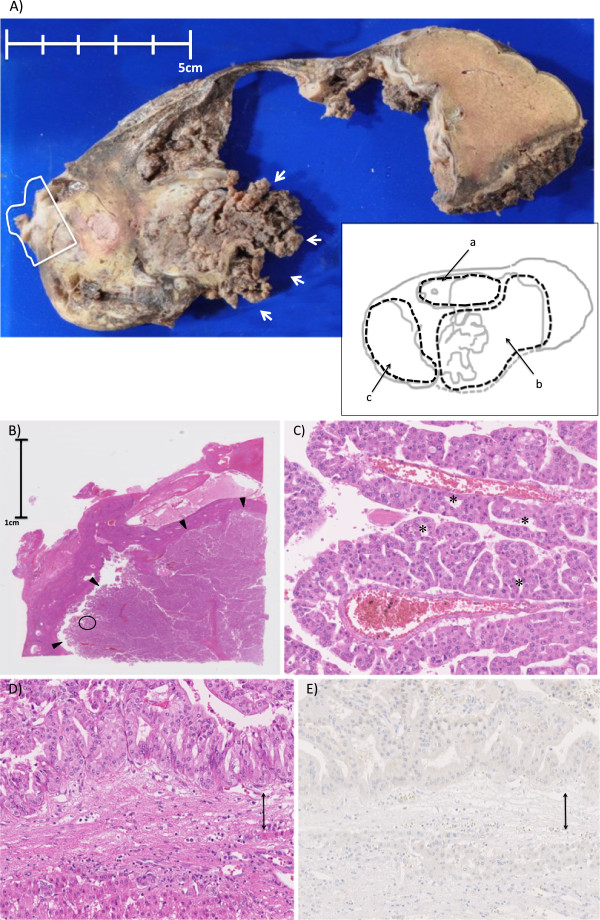
**Macroscopic and microscopic findings of the resected specimen (case 1). (A)** The gross appearance of the tumor. Macroscopically, the whole lesion was 130 x 90 mm in size in the resected specimen. The image of the lesion is illustrated as an inset in the lower right. The letters (a), (b), and (c) denote separated cystic lesions and correspond to those in Figure 1B. The medial side of the cyst wall of cyst (b) and the septum between the cysts (a) and (b) are lost in the specimen. There is a papillary mass in the posterior side of lesion (b) (indicated by arrows). The lesion (c) is filled with solid components. **(B)** A loupe observation of the solid component surrounded by a white line in **(A)** (hematoxylin and eosin). A papillary nodular mass (indicated by arrowheads) is seen inside of the cyst wall. **(C)** A magnified observation of the point encircled in **(B)** (x20, hematoxylin and eosin). Arborizing papillae with thin**-**walled vessel are seen. The lesion is lined by multilayers of cuboidal to columnar cells with abundant eosinophilic cytoplasm forming intraepithelial lumina (some of the lumens are indicated by asterisks (*)). The tumor cells had sufficient cytoarchitectural atypia to be classified as having high-grade dysplasia. **(D) (E)** A magnified (x20) image of the cyst wall (hematoxylin and eosin in **(D)**) and immunohistochemical staining for progesterone receptor in **(E)**. The cyst wall is indicated by a double arrow. Tumor components consisting of multilayer cuboidal to columnar cells (upper side of the cystic wall in the picture, inside of the tumor) and normal hepatocytes (lower side of the cystic wall in the picture, outside of the tumor) are seen. No ovarian-like stromata were seen throughout the cyst walls.

Macroscopically, the cystic lesion, 130 by 90 mm in size, was multilocular and had a solid lesion inside (Figure 2A) with a large amount of mucin. Whole liver specimens were cut into 5 to 10 mm thick pieces, which were then sliced into 5-μm sections. Histologically, the tumor showed papillary nodular growth in several cysts (Figure 2B). The tumor had complex and arborizing papillae with thin-walled vessels. The papillae or cysts were lined by multilayers of cuboidal to columnar cells with abundant eosinophilic cytoplasm forming intraepithelial lumina. The neoplastic cells had round, large, and fairly uniform nuclei with single prominent nucleoli. There was no evidence of stromal invasion; however, the tumor had sufficient cytoarchitectural atypia to be classified as having high-grade dysplasia (Figure 2C). All sections were examined carefully, but no OS were seen throughout the cyst walls (Figure 2D, E). BDC was also not evident throughout the specimens. Immunohistochemical analyses revealed that neoplastic cells were positive for MUC-5 AC and MUC-6 but not for MUC-1, MUC-2, or CDX-2. Histological and immunohistochemical findings both suggested that the tumor could be classified histologically as the oncocytic type. Since both OS and BDC were not detected in the specimen and ERC was not employed in this case, we diagnosed the cystic tumor as biliary cystadenoma, based on the WHO classification system from 2000 [1].

### Case 2

**Figure 3:**
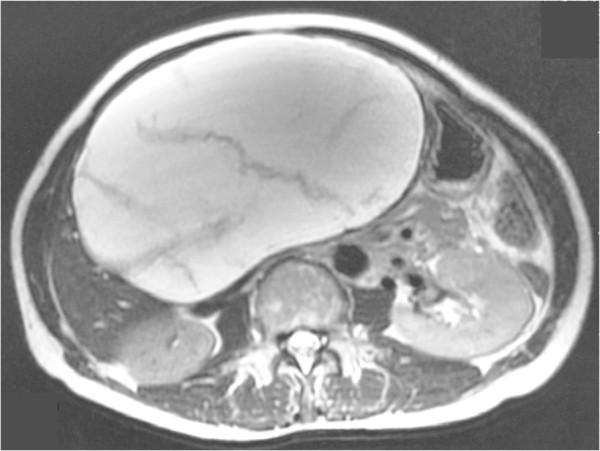
**Preoperative findings of T2-weighted magnetic resonance imaging (case 2).** A cystic lesion, 17 cm in diameter, occupies almost half of the abdominal cavity on the slice shown. Septum-like structures are seen in the lesion.

**Figure 4:**
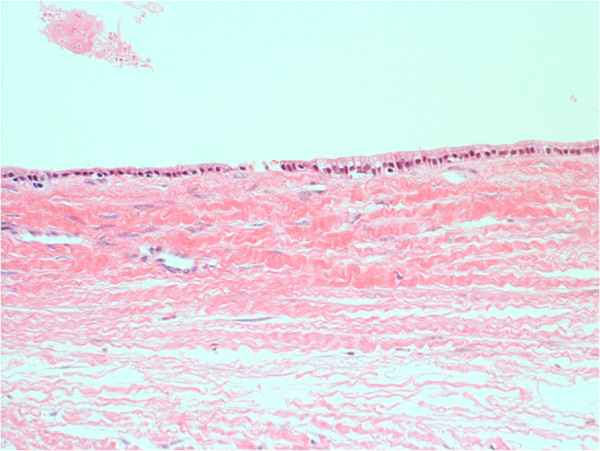
**Microscopic findings of the resected specimen (hematoxylin and eosin, x20) (case 2).** The cyst wall is lined by low columnar epithelia with slight cellular atypia. No ovarian-like stromata were seen throughout the cyst walls.

A 60-year-old Japanese woman came to our hospital in 2004 complaining of abdominal discomfort. Computed tomography with contrast enhancement revealed a unilocular cystic lesion, 17 cm in diameter, in the left lobe of the liver preoperatively. No nodular components were identified in the cystic lesion and no contrast enhancement was observed in the cyst wall. T2-weighted magnetic resonance imaging revealed septum-like structures in the cystic lesion (Figure 3). Ultrasonography also showed floating septum-like structures in the lesion. ERC was not scheduled. She had been afebrile before the operation. The white blood cell count and levels of C-reactive protein, carcinoembryonic antigen, and CA19-9 were within normal limits. Based on this preoperative information, the cystic lesion was diagnosed as a simple cyst or biliary cystadenoma and the attending surgeon scheduled an operation for cyst fenestration or left lobectomy. At the beginning of the operation, the surgeon punctured the cyst and found that the fluid was turbid brown. Although the total bilirubin concentration in the fluid was 2.1 mg/dl and cytological examination showed no atypical or malignant cells, the surgeon worried about the possibility of infection in the lesion and its spread to the abdominal cavity, so a left lobectomy was performed. The postoperative course was uneventful and the patient was discharged on postoperative day 16. The patient remains alive at nine years postoperatively.Macroscopically, the cystic lesion, 200 by 150 mm in size, was unilocular, had no solid lesion inside, and did not contain mucin. Whole liver specimens were cut into 5 to 10 mm thick pieces, which were then sliced into 5-μm sections. Histologically, the cyst wall was lined by low columnar epithelia with slight cellular atypia (Figure 4). All sections were examined at the time of surgery, but no OS or BDC were seen throughout the specimens. We diagnosed the cystic tumor as biliary cystadenoma based on the WHO classification system from 2000. We carefully re-examined the whole specimens again and confirmed that there were no OS or BDC throughout the specimens.

## Discussion

We encountered a case of cystic neoplasm of the liver in which both OS and BDC could not be pathologically confirmed (case 1). According to the most recent WHO classification, cystic neoplasms of the liver are classified as MCN with OS and without BDC, and as IPN-B with BDC and without OS [4]; therefore, it is difficult to clearly classify the case as either of these two disease entities. We assumed that the combined absence of both OS and BDC would be possible in mucinous cystic tumors of the liver, and we surveyed other cases of cystic neoplasm of the liver with mucinous epithelium that we had seen over the past 10 years at our hospital. There were three cases of cystic neoplasm of the liver with mucinous epithelium excluding the first case. They were diagnosed as biliary cystadenoma in two cases and biliary cystadenocarcinoma in one case, according to the WHO classification system in 2000 [1]. We pathologically re-examined specimens from these three cases; we confirmed the presence of OS and absence of BDC in two cases (one case of biliary cystadenoma and one case of biliary cystadenocarcinoma), and we found a case without either OS or BDC (case 2). We believe that there may be potential cases of cystic neoplasm of the liver with mucinous epithelium in which both OS and BDC are absent.

In a search of the PubMed database, we found 18 articles that addressed cystic IPN-B [5-22]. The 18 articles reported a total of 193 cases of cystic IPN-B in which BDC was confirmed in 120 cases, unclear in 11 cases, and not described in 62 cases. The 11 cases in which BDC was unclear were reported in three articles [5-7]. Kubota and associates reported 119 cases of IPN-B in their multi-institutional retrospective study and found seven cases of possible IPN-B without either OS or BDC even after their re-examination of the original sections in 2013 [5]. In 2011, Lim and associates surveyed 87 cases with surgically resected IPN-B and identified two cases in which both OS and BDC were not detected [6]. In the same year, Mano and associates reported two cases of cystic tumors of the liver without OS and BDC as possible MCN [7]. Combining the past 11 cases and our two cases, there have been 13 published cases of cystic neoplasm of the liver without OS and BDC.

We assume that there are two possibilities for cases with absence of both OS and BDC. One possibility is a failure to prove the presence of BDC during the diagnostic process. Presence of BDC is usually confirmed by preoperative ERC. However, BDC is often unclear in preoperative imaging examinations. If the BDCs are very thin and are filled with mucus, contrast medium may not flow into the communicating bile duct. There may be cases of iodine hypersensitivity as in the present case. Even in pathological examinations, it is possible that BDC cannot be detected throughout the specimen though it may in fact exist. It has been noted that intraoperative injection of contrast medium into the specimen may be technically difficult because resected liver specimens have many transected bile duct branches at the margins [7]. Another possibility is that all or some of the 13 cases of cystic neoplasm of the liver without both OS and BDC consist of a disease entity that is neither IPN-B nor MCN. Since Kubota and colleagues [5] provided little information regarding the clinicopathological features of subjects in their study, we summarized the features of four cases previously reported by Lim [6] and Mano [7] and two previous cases from our hospital (Table 1). The mean age was 57.8 years and the cohort consisted of three males and three females. The mean diameter was 13.5 cm and the diameter was greater than 10 cm in five of six cases. The tumor location was the left lobe in four cases, central bi-segment in one case, and right lobe in one case. There were two cases of malignancy and four cases of low- and/or high-grade dysplasia. It remains to be determined whether the features of middle- to old-age, no gender deviation, relatively large size, left lobe dominance, and malignant potential represent a disease entity that is neither IPN-B nor MCN. Further assessment of additional cases of cystic neoplasm of the liver without OS and BDC is needed to clarify the features of this potential disease entity.

**Table 1:** Literature review for the cystic tumor of the liver without ovarian-like stroma or bile duct communication

**Case no. [ref.]**	**Age (years)/sex**	**Diameter (cm)**	**Location**	**Ovarian-like stroma**	**Bile duct communication**	**Preoperative endoscopic retrograde cholangiography**	**Surgery**	**Pathological findings**	**Locular type**	**Survival after surgery**
1 [7]	52/F	3.2	Segments 3 and 4	(-)	(-)	(+)	Lateral segmentectomy	Low-grade dysplasia	Multilocular	Alive without recurrence 1 year
2 [7]	67/M	18	Central bi-segment	(-)	(-)	(+)	Left trisegmentectomy	Low- and high-grade dysplasia	Unilocular	Not described
3 [6]	76/M	11	Left lobe	(-)	(-)	Not described	Not described	Carcinoma	Multilocular	Not described
4 [6]	51/M	17	Left lobe	(-)	(-)	Not described	Not described	Carcinoma	Not described	Alive without recurrence 7 years
Present Case 1	41/F	15	Right lobe	(-)	(-)	(-) Iodine hypersensitivity	Right trisegmentectomy	High-grade dysplasia	Multilocular	Alive without recurrence 19 months
Present Case 2	60/F	17	Left lobe	(-)	(-)	(-)	Left lobectomy	Low-grade dysplasia	Unilocular	Alive without recurrence 9 years

## Conclusion

We presented two cases of cystic neoplasm of the liver without OS and BDC and identified four similar controversial cases from the PubMed database. Considering these six cases, we conclude that it is difficult to clearly classify cystic neoplasms of the liver with mucinous epithelium into MCN or IPN-B based only on clinical or histological findings. It is important to recognize that the most recent WHO classification is based on histological classification alone, which is probably unsatisfactory. We agree with the prevailing consensus that the presence of OS or BDC has become an important differential diagnostic criterion for IPN-B and MCN, but look forward to a better means for diagnosing cystic neoplasm of the liver in cases where both OS and BDC are not detected during preoperative examination and analysis of resected specimens. We hope that this case presentation and literature review serve as a stimulus for further investigation to establish a method to accurately diagnose cystic neoplasms of the liver with absence of OS and BDC.

## Consent

Written informed consent was obtained from the patients for publication of the case report and any accompanying images.

## Abbreviations

BDC: Bile duct communication; ER: Estrogen receptor; ERC: Endoscopic retrograde cholangiography; IPN-B: Intraductal papillary neoplasm of the bile duct; MCN: Mucinous cystic neoplasm; OS: Ovarian-like stroma; PgR: Progesterone receptor; WHO: World Health Organization.

## Competing interests

The authors declare that they have no competing interests.

## Authors’ contributions

NK wrote the manuscript. MS supervised the writing of the manuscript. YM, YF-N, and MS analyzed the pathological specimens and wrote the pathological description. OI supervised the writing of the manuscript, especially the Discussion section. AU and AT prepared the radiological images and wrote the radiological description. MK, TH, YA, and HY prepared the data from the reviewed articles. MT participated in the operation as a chief surgeon and supervised the writing of the manuscript. YK represents our surgical department and supervised the writing of the manuscript. All authors significantly contributed to this study and approved the final manuscript.
